# Mifepristone Treatment Promotes Testicular Leydig Cell Tumor Progression in Transgenic Mice

**DOI:** 10.3390/cancers12113263

**Published:** 2020-11-04

**Authors:** Donata Ponikwicka-Tyszko, Marcin Chrusciel, Kamila Pulawska, Piotr Bernaczyk, Maria Sztachelska, Peilan Guo, Xiangdong Li, Jorma Toppari, Ilpo T. Huhtaniemi, Slawomir Wołczyński, Nafis A. Rahman

**Affiliations:** 1 Department of Biology and Pathology of Human Reproduction, Institute of Animal Reproduction and Food Research, Polish Academy of Sciences, 10-748 Olsztyn, Poland; d.ponikwicka-tyszko@pan.olsztyn.pl (D.P.-T.); m.sztachelska@pan.olsztyn.pl (M.S.); slawomir.wolczynski@umb.edu.pl (S.W.); 2 Institute of Biomedicine, Research Centre for Integrative Physiology and Pharmacology University of Turku, 20520 Turku, Finland; marcin.chrusciel@orionpharma.com (M.C.); kamila.pulawska@utu.fi (K.P.); jortop@utu.fi (J.T.); 3 Department of Medical Pathomorphology, Medical University of Bialystok, 15-269 Bialystok, Poland; piotr.bernaczyk@umb.edu.pl; 4 College of Biological Sciences and Technology, Beijing Forestry University, Beijing 100083, China; guopeilan@bjfu.edu.cn; 5 State Key Laboratory of the Agro-Biotechnology, College of Horticultural Science, China Agricultural University, Beijing 100193, China; xiangdongli@cau.edu.cn; 6 Department of Reproduction and Gynecological Endocrinology, Medical University of Bialystok, 15-276 Bialystok, Poland; 7 Department of Pediatrics, Turku University Hospital, 20520 Turku, Finland; 8 Institute of Reproductive and Developmental Biology, Imperial College London, London W12 0NN, UK; ilpo.huhtaniemi@imperial.ac.uk

**Keywords:** leydig cell tumor, mifepristone, progesterone, progesterone receptors, TGFβ, PGRMC1

## Abstract

**Simple Summary:**

Recently, the antiprogestin activity of selective progesterone receptor (PR) modulator mifepristone (MF) has proven unsuccessful as a potential anti-cancer agent in various clinical trials. Herein, we analyzed the effects of MF treatment on Leydig cell tumor (LCT) progression in a transgenic mouse model (inhibin-α promoter-driven SV40 T-antigen), as well as on the proliferation of two Leydig tumor cell lines. MF significantly stimulated the proliferation of LCT in vitro. Similarly, a 1-mo MF or P4 treatment stimulated LCT tumor growth in vivo. Only the abundant membrane Pgrmc1 expression was found in LCTs, but no other classical Pgr or nonclassical membrane PRs. Functional analysis showed that PGRMC1 is required for MF and P4 to stimulate the proliferation and invasiveness of LCTs. Our findings provide novel information that the use of MF as an anti-cancer agent should be considered with caution due to its potential PGRMC1 tumor-promoting pathway activation in cancers.

**Abstract:**

The selective progesterone receptor modulator mifepristone (MF) may act as a potent antiproliferative agent in different steroid-dependent cancers due to its strong antagonistic effect on the nuclear progesterone receptor (PGR). Hereby, we analyzed the effects of MF treatment on Leydig cell tumor (LCT) progression in a transgenic mouse model (inhibin-α promoter-driven SV40 T-antigen), as well as on LCT (BLTK-1 and mLTC-1) cell proliferation. MF significantly stimulated the proliferation of LCT in vitro. Similarly, a 1-mo MF or P4 treatment stimulated LCT tumor growth in vivo. Traceable/absent classical Pgr or nonclassical membrane PRs α, β, γ and Pgrmc2, but abundant membrane Pgrmc1 expression, was found in LCTs. MF did not activate glucocorticoid or androgen receptors in LCTs. Functional analysis showed that PGRMC1 is required for MF and P4 to stimulate the proliferation and invasiveness of LCTs. Accordingly, MF and P4 induced PGRMC1 translocation into the nucleus and thereby stimulated the release of TGFβ1 in LCT cells. MF and P4 treatments upregulated *Tgfbr1*, *Tgfbr2,* and *Alk1* expression and stimulated TGFβ1 release in LCT cells. Our findings provide novel mechanistic insights into the action of MF as a membrane PR agonist that promotes LCT growth through PGRMC1 and the alternative TGFβ1 signaling pathway.

## 1. Introduction

Mifepristone (MF, RU486), classified as a selective progesterone receptor (PR) modulator (SPRM) shows strong antagonistic activity on the nuclear progesterone receptor (PGR), but depending on different PGR isoforms may also act as an agonist [1]. Recently, the antiprogestin activity of MF has proven unsuccessful as a potential anti-cancer agent in various clinical trials (such as ovarian, breast, nervous system, prostate, ovarian, and bone cancers) [2,3,4,5,6,7]. On the contrary, MF has been shown to significantly inhibit the growth of cancer cells in vitro with different PGR expression profiles [8]. The actions of progesterone (P4) may be mediated by PGRs in the genomic way, but also through mPRs α, β and γ, as well as PGRMC1 and PGRMC2 in a rapid non-genomic way [9]. The PR type that may be involved in mediating the MF effect in different cancers still remains unknown [8,10,11,12]. Recently, we have shown that MF and P4 could induce similar agonistic effects in ovarian cancer in the absence of classical PRs. Moreover, we showed MF treatment of ovarian cancer was ineffective due to its agonistic PGRMC1 action that enhanced the tumor growth [13].

Testicular tumors account for 1% of all tumors in males [14], although they are a common malignancy in men between 15 and 35 years of age [15,16]. Approximately up to 3% of all testicular tumors are believed to represent Leydig cell tumors (LCTs) [17], although a recent study showed that LCTs are more frequent than generally believed and associated with male infertility, cryptorchidism, and gynecomastia [18]. LCTs are usually clinically benign, but about 10% of the reported cases reveal a malignant phenotype [19]. LCTs have been shown to secrete steroids that can locally regulate tumor growth [20,21]. Although the expression of PGRs in the male reproductive system has been demonstrated [22] the exact role of P4 in the regulation of testicular function is still poorly understood. Early studies demonstrated expression of nuclear PGRs in rat Leydig cells (LCs) [23,24]. In human testis, PGRs have been detected in LCTs and LCs hyperplasia, as well as in traceable amounts in normal LCs [25,26,27]. Recently, it has also been reported that P4 with transforming growth factor β1 (TGF-β1) may increase cell proliferation of mouse LCs [28]. Additionally, P4 stimulates steroidogenic acute regulatory protein (StAR) expression in MA-10 cells [29]. In mouse LCTs (mLTC-1) cell line, P4 significantly inhibited luteinizing hormone receptor (LHR) expression and function. Presumably it was through their membrane PR (mPR), as mLTC-1 cells did not express classical PGRs [24,30]. Interestingly, the disruption of α and β PGR isoforms did not affect male fertility [31,32], which may suggest a non-classical P4 pathway activation in LCs. Therefore, further studies are needed to analyze the P4-PR interaction and P4 signaling pathways involved in the regulation of normal and tumorous LC function.

In the present study, we took advantage of P4 and MF treatments in a transgenic mouse model expressing Simian Virus 40 T antigen under the inhibin α promoter (Inhα/Tag) that develops endocrinologically active LCTs by five months of age with 100% penetrance [33,34]. The onset of LCTs in Inha/Tag TG mice corresponded with increased serum levels of P4, decreased gonadotropin concentrations, and an increased number of P4-secreting tumor cells in the gonads [33,35]. For in vitro MF/P4 treatment experiments, we used an immortalized murine LCT cell line (BLTK-1) derived from the Inhα/Tag TG mice and another murine LCT cell line (mLTC-1) [36]. Our goal was to study the molecular mechanisms underlying the MF and P4 action on LCTs and to characterize their nuclear and membrane PR expression profiles, as well as the MF pharmacokinetics in vivo and the MF metabolite effects on LCT proliferation in vitro.

## 2. Results

### 2.1. MF Achieves Low µM Serum Concentrations in Inhα/Tag Mouse Serum

Earlier pharmacological studies on MF have shown that MF and its metabolites (N-demethyl, Di-demethyl, and 22-hydroxy MF) achieve only a low μM serum concentrations in humans [37,38]. We evaluated the levels of MF and its metabolites in Inhα/Tag TG mouse serum. The MF pharmacokinetics was measured following a single-dose i.p. injection of 1 mg/kg or 10 mg/kg MF. The peak MF concentrations after 4 h reached 0.024 µM and 0.32 µM following the 1 mg/kg or 10 mg/kg doses, respectively (Figure S1A,B). MF was metabolized by hydroxylation and demethylation. The peaks of hydroxylated MF, mono- and di-demethylated MF were observed at 5 hours after injection and achieved 0.04, 0.009, and 0.015 µM, and 0.35, 0.135, and 0.218 µM for the 1 mg/kg or 10 mg/kg doses, respectively.

### 2.2. MF and P4 Treatmenst Affect Leydig Tumor Cell Proliferation In Vitro

**Figure 1:**
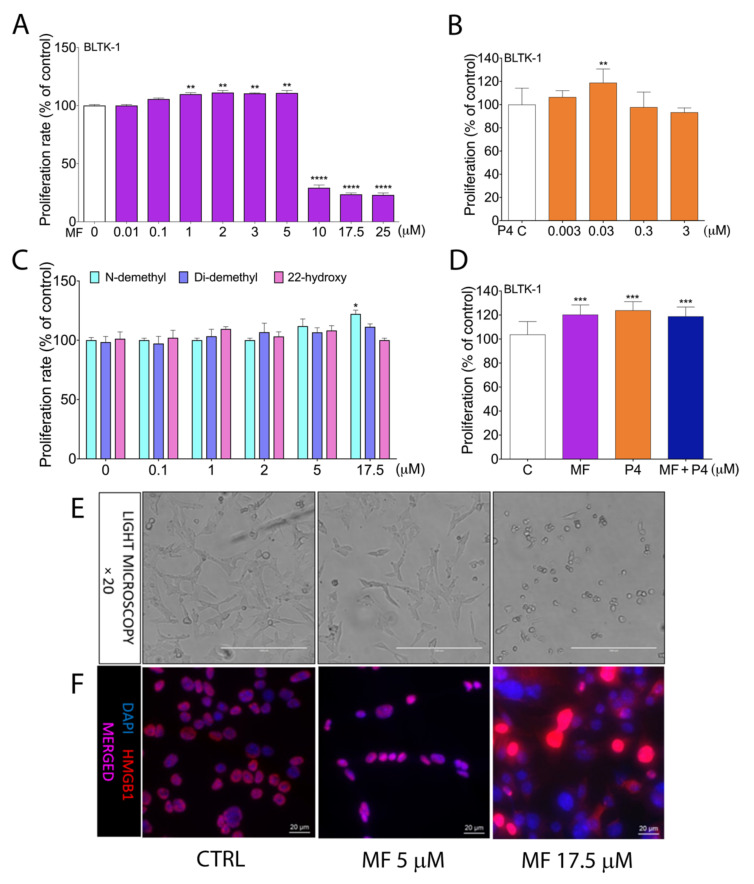
Proliferation of BLTK-1 cells with MF, P4 or MF metabolite treatments. Effects of MF (**A**), P4 (**B**), the 22-hydroxy, N-demethyl and Di-demethyl MF metabolites (**C**) and MF with P4 (**D**) on BLTK-1 cells proliferation after 72 h treatments, measured by MTT and BrdU assay. Light microscopy images of BLTK-1 cells after 5 μM or 17.5 μM MF treatment (**E**). Immunolocalization of HMGB1 protein after 5 μM or 17.5 μM MF treatment of BLTK-1 cells (**F**). The proliferation level of the treated groups is presented as the percentage of control proliferation, considered as 100%. Asterisks indicate significant differences between the control and treated groups (*, *p* < 0.05; **, *p* < 0.01; ***, *p* < 0.001; ****, *p* < 0.0001.). Scale bar, 20 μm. Di-demethyl MF, (11β,17β)-11-(4-Aminophenyl)-17-hydroxy-17-(1-propyn-1-yl)-estra-4,9-dien-3-one; 22-hydroxy MF, (11β,17β)-11-[4-(Dimethylamino)phenyl]-17-hydroxy-17-(3-hydroxy-1-propyn-1-yl)-estra-4,9-dien-3-one; Inhα/Tag mice; transgenic mice expressing the SV40 Taq oncogene under the inhibin α promoter; MF, mifepristone; N-demethyl MF, (11β,17β)-17-Hydroxy-11-[4-(methylamino)phenyl]-17-(1-propyn-1-yl)-estra-4,9-dien-3-one; P4, progesterone.

We analyzed the effects of MF (0.01–25 µM concentration) and P4 (0.003–3 µM concentration) on the proliferation of two independent Leydig tumor cell lines, BLTK-1 and mLTC-1. Low levels, up to 5 µM of MF (Figure 1A, Figure S2A), as well as 0.03 µM of P4 in BLTK-1, and 0.03 µM and 0.3 of P4 in mLTC-1, increased cell proliferation (Figure 1B, Figure S2B), whereas the higher doses of MF, but not of P4, significantly inhibited the cell proliferation in both cell lines. N-demethyl MF at a concentration of 17.5 µM significantly stimulated cell proliferation, whereas the other MF metabolites (Di-demethyl and 22-hydroxy MF) did not affect the BLTK-1 cell proliferation (Figure 1C). We also found that MF treatment did not inhibit the P4-enhanced proliferation of BLTK-1 cells (Figure 1D).

Furthermore, we found that the translocation of the cell death marker protein HMGB1 from the nucleus to the cytoplasm occurred at 17.5 µM MF, but not at 5 µM MF (Figure 1E,F), proving that the lower doses of MF do not induce cell death.

### 2.3. MF and P4 Stimulate Leydig Cell Tumor Growth In Vivo

**Figure 2:**
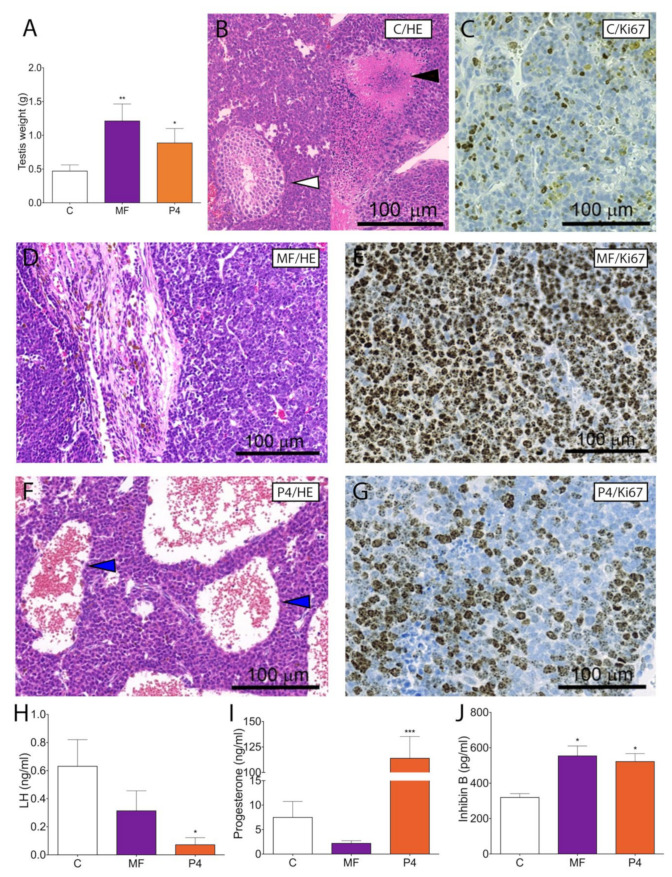
Treatment of Inhα/Tag TG mice presenting Leydig cell tumors and post-treatment hormonal values. Total testicular tumor weights (mean ± SEM) of control, MF-treated and P4-treated Inhα/Tag TG mice (**A**). Analyses of the control histopathology (**B**), control Ki-67 staining (**C**) MF-treated histopathology (**D**), MF-treated Ki-67 staining (**E**) P4-treated histopathology (**F**) and P4-treated Ki-67 staining (**G**) Inhα/Tag TG mice. Serum concentrations (mean ± SEM) of LH (**H**), P4(**I**), and inhibin B (**J**) of the non-treated (vehicle) (control), MF-treated and P4-treated Inhα/Tag TG mice. White arrow heads indicate tubular structure (**B**), black arrow heads necrotic area (**B**), blue arrow heads blood-filled cavities (**F**). Asterisks indicate significant differences between non-treated and treated groups (*, *p* < 0.05; **, *p* < 0.01; ***, *p* < 0.001). Scale bar, 100 μm. C, control; Inhα/Tag TG mice; transgenic mice expressing the SV40 Taq oncogene under the inhibin α promoter; MF, mifepristone; P4, progesterone.

To analyze the effects of MF and P4 on tumor growth in Inhα/Tag TG mice we chose 10 mg/kg of MF, corresponding to the dose used in clinical trials and another dose of 1 mg/kg of P4. The Inhα/Tag TG mice treated with these doses of MF and P4 shown increased testis weights (Figure 2A).

Histopathological analyses demonstrated in non-treated LCTs severe cellular atypia, only a few peripheral tubular structures with spermatogenic cells up to elongated spermatids and in some regions rapid tumor growth with necrosis (Figure 2B). The P4- and MF-treated LCTs showed overall destroyed histological morphology with blood-filled cavities, infiltrating lymphocytes, and with almost no normal testicular structures left (Figure 2D,F). We confirmed LCT progression after MF and P4 treatment by increased Ki67-positive cells to 60–80% vs. 40% in the non-treated group (Figure 2C,E,G).

We also analyzed the hormonal profiles after the treatments. P4 (1 mg/kg) treatment significantly decreased LH (Figure 2H) and increased serum P4 levels (Figure 2I). Both treatments increased serum inhibin B level (Figure 2J). Additionally, MF and P4 down-regulated the *Lhcgr* expression level (Figure S3).

### 2.4. MF and P4 Stimulate Leydig Cell Tumors Proliferation and Invasiveness through Pgrmc1, Independently of Their Glucocorticoid Receptors

**Figure 3:**
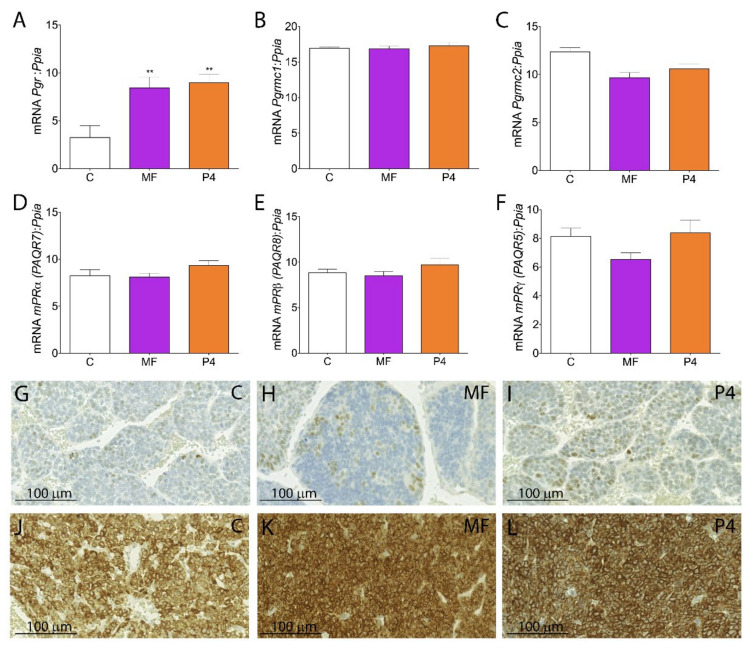
Pgr gene profiling in MF- and P4-treated transgenic Inhα/Tag TG mice and BLTK-1 cells proliferation and invasion. qPCR analysis of *Pgr* (**A**), *Pgrmc1* (**B**), *Pgrmc2* (**C**), *Paqr7* (mPRα) (**D**), *Paqr8* (mPRβ) (**E**), and *Paqr5* (mPRγ) (**F**) expression in the non-, MF- and P4-treated tumors of Inhα/Tag TG mice. Each bar represents the mean ± SEM relative to *Ppia.* Immunohistochemical staining of PGR in the control (**G**), MF-treated (**H**) and P4-treated (**I**) tumors and of PGRMC1 in control (**J**), MF-treated (**K**) and P4-treated (**L**) LCTs of Inhα/Tag TG mice. Scale bar, 100 μm. Asterisks indicate significant differences between the control and treated groups (**, *p* < 0.01). C, control; Inhα/Tag TG mice; transgenic mice expressing the SV40 Taq oncogene under the inhibin α promoter; LCT, Leydig cell tumor; MF, mifepristone; P4, progesterone.

We have characterized the expression profile for all PRs in BLTK-1 and mLTC-1 cells as well as Inhα/Tag LCTs (Figure 3A–L, Figure S4, and Table S1). The *Pgr* expression level was low in non-treated LCTs (Figure 3A).

The MF and P4 treatments significantly increased *Pgr* expression in LCTs (Figure 3A) but did not affect the expression of any of the membrane PR (*Paqr5, Paqr7, Paqr8, Pgrmc1*, *Pgrmc2*) (Figure 3B–F). Immunohistochemical studies demonstrated a weak expression of Pgr (Figure 3G–I) but abundant Pgrmc1 in non-treated, MF-, and P4-treated LCTs (Figure 3J–L). BLTK-1 cells expressed all types of PRs at the mRNA level, however the expression of PGR and mPRγ at the protein level was weak (Figure S4, Table S1). No Pgr and weak mPRγ expression was detected in mLTC-1 cells (Figure S4, Table S1).

**Figure 4:**
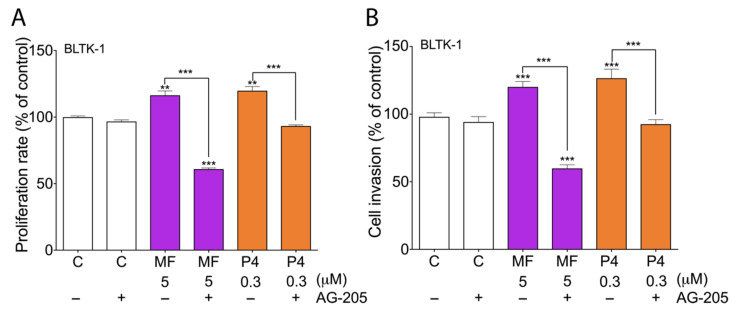
Proliferation of BLTK-1 cells with MF, P4 or PGRMC1 inhibitor treatments. Effects of MF and P4 with or without the AG-205 inhibitor on BLTK-1 cell proliferation after 24 h treatment, measured by BrdU assay (**A**). Cell proliferation of the treated groups is presented as the percentage of the control (considered as 100%). Effects of MF and P4 with or without the AG-205 inhibitor on BLTK-1 cell invasion after 24 h treatment (**B**). Cell invasion of the treated groups is presented as the percentage of the control group (considered as 100%). Asterisks indicate significant differences between the control and treated groups (**, *p* < 0.01; ***, *p* < 0.001). AG-205, PGRMC1 inhibitor; C, control; LCT, Leydig cell tumor; MF, mifepristone; P4, progesterone.

To analyze whether PGRMC1 is involved in MF or P4 actions in LCTs, we treated BLTK-1 cells with the PGRMC1 inhibitor AG-205, which inhibited both MF- and P4-stimulated BLTK-1 cells proliferation (Figure 4A).

**Figure 5:**
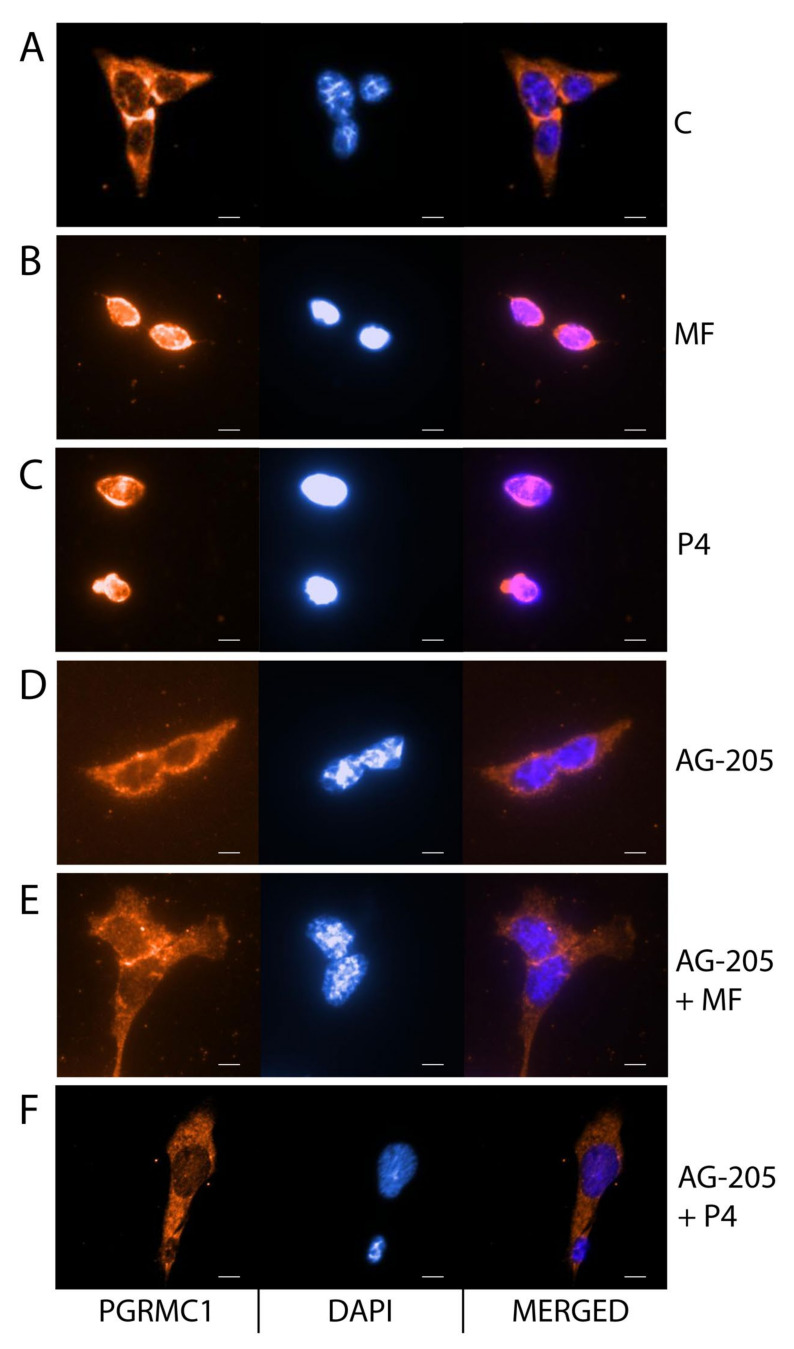
MF and P4 treatments effects on the nuclear translocation of PGRMC1 in BLTK-1 cells. Immunocytochemical localization of PGRMC1 without C (**A**) or with MF (**B**), P4 (**C**), AG-205 (**D**), AG-205 + MF (**E**) and AG-205 + P4 (**F**) in BLTK-1 cells. Scale bar, 20 μm. AG-205, PGRMC1 inhibitor; C, control; MF, mifepristone; P4, progesterone.

The PGRMC1 inhibitor cotreatment significantly decreased BLTK-1 cell proliferation compared to the control group (Figure 4A). Moreover, the PGRMC1 inhibition significantly reduced MF- and P4-induced BLTK-1 cell invasion (Figure 4B). We also studied the nuclear translocation of PGRMC1 after the MF and P4 treatments in BLTK-1 cells (Figure 5A–F). MF and P4 treatments induced the translocation of PGRMC1 to the nucleus (Figure 5B,C), whereas PGRMC1 blockage abolished PGRMC1 nuclear translocation in BLTK1 cells (Figure 5D–F).

As MF may also bind to glucocorticoid receptors (GR), we assessed the *Gr* and GR-target gene expression levels [39,40,41] and checked the immunolocalization of Gr after MF treatment in BLTK-1 cells (Figures S5A–F and S6A–D). Neither MF nor P4 treatments affected the expression of *Gr* (Figure S5A,B) or its target genes *Fkbp5*, *Ska2*, *Oct1,* and *Oct2* (Figure S6A–D). Immunocytochemistry demonstrated that Gr did not show nuclear staining after MF or MF with the Gr inhibitor (HSPi90) treatments (Figure S5E–F). A positive control, dexamethasone (DXM), induced Gr nuclear translocation (Figure S5D).

We also checked the MF and P4 treatment effects on androgen receptor (*Ar*) expression level, which was not affected by in vivo treatments in the Inhα/Tag TG mouse LCT or in vitro in BLTK-1 cells (Figure S7A,B).

We finally assessed the Simian Virus 40 T antigen (SV40 Tag) expression levels before and after the MF and P4 treatments in the Inhα/Tag TG mouse LCT and BLTK-1 cells, to exclude the potential interaction of the oncogene with MF and P4 actions in LCTs (Figure S8A,B). The mRNA level of *SV40 Tag* did not change after the MF and P4 treatments in LCTs (Figure S8A), or after the MF, P4, and PGRMC1 inhibitor AG-205 treatments in LCT cells (Figure S8B).

### 2.5. MF and P4 Promote Tumor Progression through Activation of the TGFβ1 Superfamily Signaling Pathway

**Figure 6:**
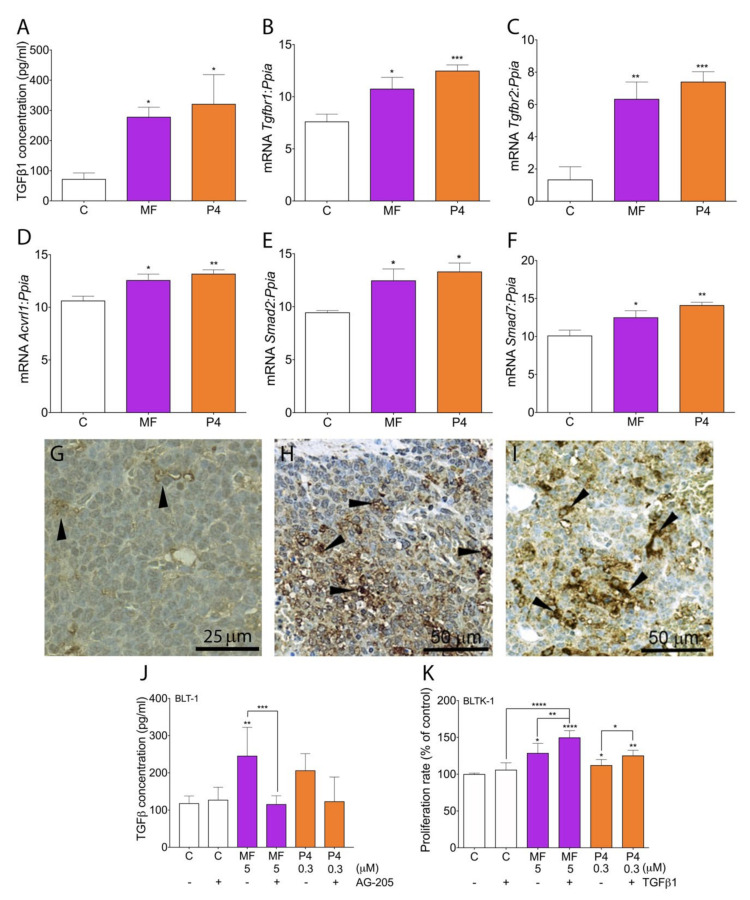
MF and P4 treatments effects on TGFβ1 superfamily signaling pathways. TGFβ1 serum level in the control, MF-treated or P4-treated tumors of Inhα/Tag TG mice (**A**). qPCR analysis of the *Tgfbr1* (**B**)*, Tgfbr2* (**C**)*, Acvrl1 (Alk1)* (**D**), *Smad2* (**E**), and *Smad7* (**F**) expression levels in the control, MF-treated and P4-treated tumors of Inhα/Tag TG mice. Each bar represents the mean ± SEM relative to *Ppia.* Immunohistochemical staining of TGFβR2 in the C (**G**), MF-treated (**H**) and P4-treated (**I**) tumors of Inhα/Tag TG mice. Black arrows indicate Tgfβr2 positive staining. Scale bar, 25 μm or 50 μm. TGFβ1 level in the control, MF-treated or P4-treated BLTK-1 cells with or without the AG-205 inhibitor (**J**). Effects of recombinant TGFβ1 treatment on MF- or P4-treated BLTK-1 cells (**K**). The proliferation level of the treated groups is presented as the percentage of control proliferation, considered as 100%. Asterisks indicate significant differences between the non-treated control and treated groups (*, *p* < 0.05; **, *p* < 0.01; ***, *p* < 0.001; ****, *p* < 0.0001). AG-205, PGMC1 inhibitor; C, control; Inhα/Tag TG mice; transgenic mice expressing the SV40 Taq oncogene under the inhibin α promoter; MF, mifepristone; ND, non-detectable; P4, progesterone.

As the TGFβ1 superfamily pathway may be involved in cancer progression [42], we characterized the TGFβ1 family member expression profile in LCTs. MF and P4 treatments increased serum TGFβ1 levels in Inhα/Tag TG mice compared with non-treated mice (Figure 6A).

Both the MF and P4 treatments significantly up-regulated *Tgfbr1*, *Tgfbr2*, *Acvrl1, Smad2,* and *Smad7* expression level in LCTs (Figure 6B–F). The non-treated group of LCTs showed weak expression of Tgfβr2 (Figure 6G), whereas after MF and P4 treatments, LCTs showed abundant Tgfβr2 staining (Figure 6H,I). Both treatments increased the TGFβ1 release by BLTK-1 cells, whereas PGRMC1 blockage inhibited this effect (Figure 6J). The MF and P4 treatments with recombinant TGFβ1 cotreatment significantly up-regulated the proliferation of BLTK-1 cells and showed an additive effect to MF (Figure 6K).

## 3. Discussion

P4 has been shown to be produced and involved in the regulation of LC and LCT function and proliferation [24,30], suggesting that LCTs could be susceptible to antiprogestin treatment. MF has been shown to inhibit in vitro the growth of cancer cells of reproductive and non-reproductive origin, independently of their PGR expression status [8]. However, the clinical MF trial results on several human cancers have been disappointing [3,7]. This discrepancy between the experimental and clinical data prompted further studies to understand the molecular mechanisms underlying the MF action in cancers.

It has been shown that MF in vitro at concentrations of 10–100 μM inhibits cancer cell growth [8,10,11,12,43]. However, MF at the lower 1 μM concentration did not affect human ovarian epithelial cancer cells proliferation [44]. Our earlier [13] and present results show that MF may exert its effect in a biphasic way. Recently, it has also been shown that the major active MF metabolite, metapristone (N-demethyl mifepristone), inhibited cancer cell proliferation in a dose-dependent manner [45,46]. However, our in vitro experiments demonstrated that metapristone at lower doses may significantly stimulate tumor cell proliferation. Therefore, our data suggest that MF and metapristone at low levels may have a stimulatory effect on tumor cells and may not achieve sufficient tissue levels to inhibit cancer cell progression.

It has been shown that the serum concentrations of MF were not affected by increasing doses of the MF from 200 to 800 mg and reached a maximum of 2.5 μM serum concentration in all patient groups after 24h [47]. Even long-term treatments up to 20 months did not change the serum concentration of MF [48]. The dose of 10 mg/kg of MF in mice corresponds to the highest dose used in human clinical trials [13]. As there were no changes in serum MF levels in humans between the doses of 200–800 mg/day, any changes after higher doses in mice were not either expected. It is highly unlikely to achieve a high level, e.g., 10 µM of MF in the tissues, due to its rapid metabolism and binding to the high-affinity binding protein alpha 1-acid glycoprotein (AAG) [49]. The serum concentration of ~2.5 μM corresponds to the plasma protein AAG binding capacity of MF [49]. Only the unbound drug, i.e., low circulating 2.5 μM of MF concentration, is available for target tissues to exert its pharmacological effects [13,49].

P4 may mediate its signal in a dual mode through genomic and non-genomic ways, although the key mode of PR action on LC and LCT function has not been described [30]. The type of PR that may be involved in mediating the MF effect in different cancers also remains unknown [8]. Expression of the classical Pgr was not observed in mLTC-1 cells [30]. Moreover, the effect of MF on the P4 action on mLCT-1 cells was inconsistent, suggesting that P4 affects LCs independently of the classical P4 signaling pathway [24]. In the present study, we characterized the expression profile of all the nuclear and membrane PRs in LCTs of Inhα/Tag TG mice, BLTK-1, mLTC-1 cells. However, only Pgrmc1 expression was high in LCTs, suggesting its functional role in these tumors. Besides the characterization of PRs, we also checked the GR activation, as MF may also affect the GR and bind to different GRs isoforms (α and β) with high affinity [37]. Recent studies of MF action on GRs are inconsistent, as one of them reported that MF may stimulate GRβ nuclear translocation, but another did not [50,51]. It has also been shown that MF rather inhibits than activates GRs action in LCs and LCTs [52,53]. We were unable to find any connections between MF and nuclear translocation of Gr or Gr-related stimulation of gene expression in BLTK-1 cells. Our data suggest that MF acts independently of GRs activation and rather mediates its action through mPRs in LCTs. Moreover, in MA-10 cells MF significantly stimulated StAR expression at a level comparable with P4 independently of Gr, indicating that the effect may be mediated through the membrane PRs pathway [29]. Our in vitro studies showed that membrane receptor PGRMC1 is required for MF and P4 to stimulate the proliferation and invasiveness of LCTs. These results indicate that MF acts as a selective membrane P4 agonist through PGRMC1 activation in LCTs. In human ovarian cancers, abundant PGRMC1 expression level has also been demonstrated [13,54]. Moreover, PGRMC1 has been involved in ovarian cancer cell invasion [13,55].

The results of this study showed that both MF and P4 may stimulate the alternative tumor-promoting TGFβ1 superfamily signaling pathway in LCTs. TGF-β1 is a member of a large cytokine family involved in many biological processes, including cell proliferation, differentiation, migration, adhesion and survival, in both normal and cancerous cells [56]. In LCs, TGF-β1 has been shown to influence steroidogenesis and regulate cell proliferation [28,57]. We also showed that MF and P4 enhanced *Acvlr1* (*Alk1*), *Smad2,* and *Smad7* expression levels. It has been suggested that TGF-β1 via the ALK1 signaling may lead to epithelial cell proliferation [58,59]. The high expression levels of TGF-β1signaling elements, especially ALK1 has also been shown in patients with LC hyperplasia [27]. Additionally, P4 with TGFβ1 has been considered as the domain factors causing LC hyperplasia/hypertrophy [28]. Morphometric testicular analyses of mice treated with P4 and TGF-β1 revealed increased volume of LCs [28]. Studies also demonstrated enhanced expression of the proliferation marker PCNA in LCs after P4 and TGFβ1 treatments. Additionally, P4 and TGFβ1 treatments reduced the expression level of the proapoptotic gene *Bax* [28]. Our present data revealed that MF and P4 also enhanced the expression of the cell-cycle progression marker Ki-67 in LCTs of Inhα/Tag TG mice. Ki67 is not a cell proliferation marker per sein the sense that it labels cells in S-Phase of the cell cycle. Ki67 labeling can be found in nuclei throughout the cell cycle, usually, except in the cells that are in the G0 phase. Ki67 shows thus that the cells have the capacity to proliferate and are not terminally differentiated. MF treatment increased also TGFβ1 release in BLTK-1 cells. PGRMC1 inhibition significantly reduced this effect, indicating that PGRMC1 is involved in MF and P4 activation of the TGFβ1 signaling pathway in LCTs. Our data suggest that, in MF and P4 action, PGRMC1 may be the key LCT P4 receptor in the tumor-promoting action of TGFβ1.

PGRMC1 expression has also been shown in several cancer cell, like in breast, prostate and lung, emphasizing the translational aspect of such findings [55,60,61]. Recently, it has been demonstrated that PGRMC1 promotes tumorigenesis, cell proliferation, migration, invasion, and antiapoptosis in the same cancer types [55,60,61,62]. Additionally, higher expression of PGRMC1 may be useful in the prediction of prognosis of breast cancer patients [63]. This issue is becoming even more important, as patients with these cancers are still being recruited for ongoing clinical trials with MF [64,65,66]. The use of MF as anti-cancer agent should be reconsidered in the light of its potential of tumor promoting action through activation of the PGRMC1 pathway.

## 4. Materials and Methods

### 4.1. Experimental Animals

In vivo studies were done on previously characterized Inhα/Tag TG mice [35]. The 5.5 months of age male mice with discernible testicular tumors were randomized into three groups (*n* = 10 mice/group) and intraperitoneally injected every 2 days either with vehicle (corn oil) or MF (10 mg/kg; Sigma-Aldrich, Saint Louis, MO, USA) or P4 (1 mg/kg; Sigma-Aldrich, Saint Louis, MO, USA) for 1 month. Mice were fed with mouse chow SDS RM-3 (Special Diet Service; E, soy free; Whitham Essex, UK), tap water ad libitum and kept in a specific pathogen-free surrounding and routinely screened for common mouse pathogens. After 30 days of treatments mice were sacrificed, blood and tissue samples were collected. Half of each tumor was fixed in 4% paraformaldehyde and embedded in paraffin for histological and immunohistochemical studies. The second half of the tumor tissue was snap-frozen in liquid nitrogen and stored at −80 °C for RT-PCR analysis. The Ethics Committee for animal experimentation of the University of Turku and the State Provincial Office of Southern Finland approved all animal experiments (Animal Licence number: ESAVI/5757/04.10.07/2017).

### 4.2. MF Pharmacokinetics

MF pharmacokinetic was analyzed in 6 mo-old Inhα/Tag male mice. Mice were intraperitoneally injected with a single dose of 1 mg/kg of MF (*n* = 5) and 10 mg/kg of MF (*n* = 5). Blood samples were collected after 30 min, 4 h, and 8 h, 16 h, 24 h, and 48 h. Concentrations of MF and its metabolites N-demethyl MF, Di-demethyl MF and 22-hydroxyl MF in mouse plasma were determined using high performance liquid chromatography–mass spectrometry (HPLC-MS/MS) after protein precipitation with internal standard alfaxalone. HPLC separation was performed with Agilent 1200 LC system, using a C18 column. Multiple-reaction monitoring with a triple quadrupole mass spectrometer was used for quantitative analyses (AB Sciex 4000 QTrap with Analyst software (v. 1.6.1); MDS Sciex, Ontario, Canada). Standards 22-hydroxy (H948445), Di-demethyl (D439550) and N-demethyl mifepristone (D230950) were bought from Toronto Research Chemicals Inc. (Toronto, Ontario, Canada).

### 4.3. Cell Cultures

The BLTK-1 cells were cultured in DMEM/F12 medium (GIBCO, Paisley, UK) supplemented with 10% fetal bovine serum (FBS; Biochrom, Berlin, Germany), 100 units/ml penicillin and 100 µg/ml streptomycin (P/S solution; Sigma-Aldrich) at 37 °C in a humidified atmosphere in the presence of 5% CO2. The mLTC-1 cells were cultured in Waymouth’s medium (GIBCO, Paisley, UK) supplemented with 10% horse serum (GIBCO, Paisley, UK) and 5% FBS (Biochrom, Berlin, Germany), and P/S solution (Sigma-Aldrich, Saint Louis, MO, USA) at 37 °C in a humidified atmosphere in the presence of 5% CO_2_.

### 4.4. Cell Proliferation

Cell proliferation was analyzed using CellTiter 96® AQueous Non-Radioactive Cell Proliferation Assay (Promega, Madison, WI, USA) and BrdU Cell Proliferation Assay Kit (Cell Signaling Technology, Danvers, MA, USA). BLTK-1 were seeded (10 × 10^3^/well) in culture medium onto 96-well plate and after 16 h treated with vehicle (EtOH 0.05%), MF (0.01; 0.1; 1; 2; 3; 5; 7; 17; 25 μM), P4 (0.003; 0.03; 0.3; 3 μM), a PGRMC1 inhibitor AG-205 (1 μM, Sigma-Aldrich, Saint Louis, MO, USA) and TGFβ1 (10 μM, 240-B; R&D Systems Inc., Minneapolis, MN, USA) in stimulation medium (phenol-free DMEM/F12 with 0.5% charcoal-stripped FBS and P/S solution) for 72 h with MF or P4 for 24 h with AG-205/TGFβ1. The proliferation rate was presented as a percentage of control proliferation considered as 100%. Three independent experiments per cell line were run, each performed in octuplicate wells.

### 4.5. Cell Invasion

Cell invasion intensity of BLTK-1 cells was assessed using CultreCoat® Cell Invasion Assays (R&D Systems, Minneapolis, MN, USA). Briefly, 2.5 × 10^4^ cells/well were transferred to each of 96-well plate top invasion chamber coated with Basement Membrane Extract (BME). Cells invaded in response to MF, P4 and AG-205 (1 μM) were quantitated using Calcein AM after 24 h of treatment. Three independent experiments were run, each performed in octuplicate wells. Cell invasion intensity of the treated groups was presented as percentage of invasion of control group, considered as 100%.

### 4.6. Histological and Immunohistochemical Analyses

Mouse testicular tumor tissues were fixed in paraformaldehyde and embedded in paraffin. For histological analysis, 5 μm paraffin sections were stained with hematoxylin-eosin. For immunohistochemistry sections were deparaffinized, hydrated and boiled in 10 mM citric acid buffer (pH 6.0) in retriever for 2.5 h. Tissue sections were incubated with blocking solutions (10% normal goat serum (NGS) with 3% bovine serum albumin (BSA) or only 3% BSA in PBS) for 1 h at room temperature in order to reduce non-specific background staining. Then, sections were incubated overnight at 4 °C with the primary antibodies for PGR (MA5-12658, Thermo Fisher Scientific Inc., Waltham, MA, USA; dilution 1:700), mPRα (ab75508, Abcam, Cambridge, UK; dilution 1:500), mPRβ (ab46534, Abcam; dilution 1:1000), mPRγ (ab79517, Abcam; Cambridge, UK; dilution 1:500), PGRMC1 (PAB20135, Abnova Corporation, Taipei, Taiwan; dilution 1:2000), PGRMC2 (ab125122, Abcam; Cambridge, UK; dilution 1:1000), TGFβRII (sc-220, Santa Cruz Biotechnology, Dallas, TX, USA; dilution 1:700), Ki-67 (Clone TEC-3, Dako, Glostrup, Denmark; dilution 1:500), IgG (ab190475, Abcam; Cambridge, UK; dilution 1:700), IgG2a (ab190463, Abcam; Cambridge, UK; dilution 1:500). After endogenous peroxidase blocking (0.5% H_2_O_2_ in PBS for 20 min in dark at room temperature) primary antibodies were linked with Envision® anti-mouse or anti-rabbit polymer + HRP (Dako, Glostrup, Denmark) for 30 min at room temperature, only for Ki-67 staining before this step, secondary antibody rabbit anti rat was added (Dako, Glostrup, Denmark; dilution 1:200). The reaction product was visualized using 3’3-diaminobenzidine tetrahydrochloride (DAB, Dako, Glostrup, Denmark). Three washes were done after each step with PBS with 0.05% Tween (PBS-T). Hematoxylin was used as counterstain and then sections were dehydrated and mounted with Pertex (Histolab Products AB, Spånga, Sweden). Control immunohistochemical stainings of the IgG2a and IgG are shown in Figure S9A–D.

### 4.7. Immunocytochemistry Analysis

BLTK-1 cells 1–2 × 10^4^ cells/well were seeded onto microscope slide coverslips and after 16 h treated with vehicle (0), MF (5 μM, 17.5 μM), vehicle (0), MF (3 μM), DXM (200 nM), MF (3 μM) + HSP90i (50 nM), HSP90i (50 nM) + DXM (200 nM) or vehicle (0), MF (3 μM), P4 (0.3 μM), AG-205 (1 μM), MF (3 μM) + AG-205 (1 μM), and P4 (0.3 μM) + AG-205 (1 μM) in stimulation medium. Cells were fixed in 4% PFA in PBS pH 7.4 for 15 min at room temperature and permeabilized for 10 min in 0.1% Triton X-100. To reduce autofluorescence cells were incubated with 100 mM NH4CL for 10 min. After blocking unspecific binding sites with 3% BSA in PBS with 0.05% Tween 20 for 30 min cells were incubated for 1 h with primary antibodies anti-GR (SC-56851, Santa Cruz Biotechnology, Dallas, TX, USA; dilution 1:400), anti-PGRMC1 (PAB20135, Abnova Corporation; dilution 1:1000) or anti-HMGB1 (ab79823, Abcam, Cambridge, UK; dilution 1:350) diluted in blocking solution. Next, cells were incubated with secondary fluorescent antibody Alexa Fluor 488 goat anti-mouse IgG (ab150113, Abcam, Cambridge, UK; dilution 1:400) or Alexa Fluor 647 donkey anti-rabbit IgG (Life Technologies, Carlsbad, CA, USA; dilution 1:600) for 45 min. To detect cell nuclei, cells were incubated with DAPI for 1 min.

### 4.8. Real Time RT-PCR

Total RNA from cells and snap-frozen LCTs were prepared using TRIzol extraction method (Invitrogen, Carlsbad, CA). The quantity and quality of isolated RNA was determined by NanoDrop (Thermo Fisher Scientific Inc., Waltham, MA, USA) and gel electrophoresis. Before the reverse transcription (RT) reaction 1 µg of total RNA was incubated for 30 min with DNase I (Invitrogen, Carlsbad, CA) at room temperature. The RT reaction was performed with DyNAmo ^TM^ cDNA Synthesis Kit (Finnzymes, Espoo, Finland) at 37 °C for 1 h in 20 µl. Quantification of investigated genes was performed with FX96™ Real-Time PCR Detection System, Bio Rad using DyNAmo SYBR Green qPCR kit (Finnzymes, Espoo, Finland). Reaction conditions were: initial denaturation at 95 °C for 10 min followed by 40 amplification cycles at 95 °C for 15 s, 56–60 °C at 45 s and 70 °C at 45 s. At the end of the PCR reaction, melting curve was determined to ensure single product amplification. Amplification products were separated on 1.8% agarose gel and stained with ethidium bromide. Expression levels were normalized to the housekeeping gene peptidylprolyl isomerase (*Ppia*). The primer sequences and expected product sizes are shown in Table S2.

### 4.9. Hormones and TGFβ1 Measurement

Serum levels of LH and FSH were measured by immunofluorometric assays (Delfia; Perkin-Elmer-Wallac, Turku, Finland) as described previously [67,68]. Serum P4 level was measured using Delfia Progesterone Kit (Wallac, Perkin Elmer, Turku, Finland). The intra- and interassay coefficients of variations for these assays were below 10%. Serum level of inhibin B was evaluated by immunoassay Inhibin-B EIA Kit (Sigma-Aldrich, Saint Louis, MO, USA). TGFβ1 level in serum and cell culture supernates was assessed using TGFβ1 Quantikine ELISA Kit (R&D Systems, Minneapolis, MN, USA), following the instructions of the manufacturer.

### 4.10. Statistical Analysis

Numerical data are presented as mean ± SEM. To analyze statistical significance one-way ANOVA with the post-hoc Bonferroni’s multiple comparison post-hoc test with 95% confidence interval was used (GraphPad PRISM v. 7. GraphPad Software Inc., San Diego, CA, USA). Results were considered to be statistically significant at *p* < 0.05 level.

## 5. Conclusions

In conclusion, based on our results, we suggest that MF in low concentration may act as a membrane PR agonist and activate through PGRMC1 the tumor progression signaling pathway of TGFβ1 superfamily in LCTs. MF may also induce the PGRMC1 nuclear translocation and increase the proliferation and invasion of LCTs. Hence, it is possible that the MF anti-tumor effects observed in many cancer cell lines may not be achievable in vivo in cancer tissues and MF might not be considered as an anti-cancer agent.

## Supplementary Materials

The following are available online at https://www.mdpi.com/2072-6694/12/11/3263/s1, Figure S1: Pharmacokinetic analysis of MF metabolism in Inhα/Tag TG mice, Figure S2: Proliferation of mLTC-1 cells with MF or P4 treatments, Figure S3: *Lhcgr* expression level in Leydig cell tumors of Inhα/Tag TG mice, Figure S4: Characteristics of progesterone receptors mRNA levels in BLTK-1 and mLTC-1 cell lines, Figure S5: MF and P4 treatments effects on glucocorticoid receptor, Figure S6: Gr-target genes expression profile in BLTK-1 cells, Figure S7: MF and P4 treatments effects on androgen receptor expression, Figure S8: *SV40 Tag* expression level in Inhα/Tag TG mice and BLTK-1 cells, Figure S9: Isotype negative control staining, Table S1: Characteristics of progesterone receptors mRNA level and immunoreactivity in murine Leydig cell tumor and murine Leydig tumor cell line, Table S2: Primer sequences for RT-qPCR.

## Abbreviations

22-hydroxy MF	(11β,17β)-11-[4-(Dimethylamino)phenyl]-17-hydroxy-17-(3-hydroxy-1-propyn-1-yl)-estra-4,9-dien-3-one
AG-205	inhibitor PGRMC1
BLTK-1	Immortalized cell line from Inhα/Tag TG mice
DXM	Dexamethasone
Di-demethyl MF	(11β,17β)-11-(4-Aminophenyl)-17-hydroxy-17-(1-propyn-1-yl)-estra-4,9-dien-3-one
GR	Glucocorticoid receptor
HMGB1	High mobility group box 1 protein
HSP90i	Glucocorticoid receptor inhibitor
Inhα/Tag	Transgenic mice expressing Simian Virus 40 T antigen under inhibin-α promoter
LC	Leydig cell
LCT	Leydig cell tumor
LHR	Luteinizing hormone receptor
MF	Mifepristone
mPR	Membrane progesterone receptor
N-demethyl MF	11β,17β)-17-Hydroxy-11-[4-(methylamino)phenyl]-17-(1-propyn-1-yl)-estra-4,9-dien-3-one
P4	Progesterone
PGR	Nuclear progesterone receptors
PGRMC1	Progesterone receptor membrane component 1
PR	Progesterone receptors
SPRM	Selective progesterone receptor modulator
StAR	Steroidogenic acute regulatory protein
TG	Transgenic
TGF-β1	Transforming growth factor β1
